# Temporal changes of cytochrome P450 (*Cyp*) and eicosanoid-related gene expression in the rat brain after traumatic brain injury

**DOI:** 10.1186/1471-2164-14-303

**Published:** 2013-05-04

**Authors:** Matthew Birnie, Ryan Morrison, Ramatoulie Camara, Kenneth I Strauss

**Affiliations:** 1 University of Cincinnati College of Medicine, 231 Albert Sabin Way ML 515, 45267 Cincinnati, OH, USA; 2 Present Address: Michigan State University College of Human Medicine, 333 Bostwick Ave NE, 49503 Grand Rapids, MI, USA

**Keywords:** Cytochrome P450 genes, Arachidonic acid metabolic genes, Hippocampus, Parietal cortex, qPCR low density array, In situ hybridization

## Abstract

**Background:**

Traumatic brain injury (TBI) induces arachidonic acid (ArA) release from cell membranes. ArA metabolites form a class of over 50 bioactive eicosanoids that can induce both adaptive and/or maladaptive brain responses. The dynamic metabolism of ArA to eicosanoids, and how they affect the injured brain, is poorly understood due to their diverse activities, trace levels, and short half-lives. The eicosanoids produced in the brain postinjury depend upon the enzymes present locally at any given time. Eicosanoids are synthesized by heme-containing enzymes, including cyclooxygenases, lipoxygenases, and arachidonate monoxygenases. The latter comprise a subset of the cytochrome P450 “*Cyp*” gene family that metabolize fatty acids, steroids, as well as endogenous and exogenous toxicants. However, for many of these genes neither baseline neuroanatomical nor injury-related temporal expression have been studied in the brain.

In a rat model of parietal cortex TBI, *Cyp* and eicosanoid-related mRNA levels were determined at 6 h, 24 h, 3d, and 7d postinjury in parietal cortex and hippocampus, where dynamic changes in eicosanoids have been observed. Quantitative real-time polymerase chain reaction with low density arrays were used to assay 62 rat *Cyp*s, 37 of which metabolize ArA or other unsaturated fatty acids; 16 eicosanoid-related enzymes that metabolize ArA or its metabolites; 8 eicosanoid receptors; 5 other inflammatory- and recovery-related genes, plus 2 mouse *Cyp*s as negative controls and 3 highly expressed “housekeeping” genes.

**Results:**

Sixteen arachidonate monoxygenases, 17 eicosanoid-related genes, and 12 other *Cyp*s were regulated in the brain postinjury (p < 0.05, Tukey HSD). Discrete tissue levels and distinct postinjury temporal patterns of gene expression were observed in hippocampus and parietal cortex.

**Conclusions:**

The results suggest complex regulation of ArA and other lipid metabolism after TBI. Due to the temporal nature of brain injury-induced *Cyp* gene induction, manipulation of each gene (or its products) *at a given time after TBI* will be required to assess their contributions to secondary injury and/or recovery. Moreover, a better understanding of brain region localization and cell type-specific expression may be necessary to deduce the role of these eicosanoid-related genes in the healthy and injured brain.

## Background

The conversion of arachidonic acid (ArA) to over 50 bioactive eicosanoids is catalyzed by a variety of enzymes including heme-containing cyclooxygenases, prostanoid synthases, lipoxygenases, and arachidonate monoxygenases. The latter, arachidonate hydroxylases and epoxygenases, comprise a subset of the cytochrome P450 or “*Cyp*” gene family of evolutionarily related proteins that metabolize polyunsaturated fatty acids, steroids, as well as endogenous and exogenous toxicant molecules in organisms from bacteria to primates [1].

These *Cyp* arachidonate monoxygenases produce hydroxyeicosatetraenoic acids and epoxyeicosatrienoic acids (HETEs and EETs) that modulate a variety of responses in the healthy and injured brain. HETEs and EETs have been implicated in the physiology of the febrile response, stimulation of hypothalamic somatostatin release, pituitary vasopressin, oxytocin and luteinizing hormone release, pancreatic glucagon and insulin release, inhibition of platelet aggregation, inhibition of the activity of Na^+^K^+^-ATPase in the nephron and corneal epithelium, regulation of blood pressure, vasodilation of local microcirculation in the kidney, intestine, heart and brain [2-5]. Their activities include cerebral vasoconstriction [6-10] and vasodilatation [11-15], possibly by modulation of monovalent and divalent ion flux [6,16-24].

Many of these *Cyp*s are induced during inflammatory challenge, however recent evidence suggests that their products may diminish inflammation [2,25-27]. A number of neuron-specific effects have also been ascribed to HETEs and EETs, e.g., 12-HETE neuroprotection from glutamate-mediated cell death [28] and 14,15-EET acceleration of axonal growth [29] in primary cell cultures. In addition, EETs recently have been implicated in central nociceptive and hyperalgesic responses, with different EETs potentially moderating different effects on pain perception [22,30-34].

Brain injuries activate phospholipases and result in the release of fatty acids such as ArA from damaged membranes [35-37]. Within 48 h of traumatic brain injury (TBI), free fatty acid levels increased in human cerebral spinal fluid (CSF), compared to neurologically unimpaired controls [38]. Arachidonic acid (20:4 ω-6) increased 1093%; docosahexenoic acid (22:6 ω-3) 475%; oleic acid (18:1) 492%; myristic acid (14:0) 279%; linoleic acid (18:2) 203%; and palmitic acid (18:0) 175%. ArA levels can remain elevated for days after TBI [36]. The eicosanoids formed in different brain regions in response to TBI change dynamically over the minutes, hours, and days postinjury, likely due to local alterations in the expression of their synthetic enzymes. Regulation of prostaglandin and leukotriene metabolites and many of their source enzymes in the brain and after TBI have been well described [39-50]. Dynamic changes in P450 eicosanoid levels also occur in the injured brain [15,41,51-57]. However, the production, enzymatic sources, and local regulation after brain injury of hydroxylases, epoxygenases and their products have been less well studied.

Local brain HETE and EET levels have not been well described due to their trace levels, short half-lives, and closely related chemical structures. Moreover, because of the historic lack of gene-specific antibodies and molecular probes, it has been difficult to characterize the regulation of individual *Cyp* genes in this large family. Completion of the sequencing of the rat genome [58] has yielded molecular probes specific for each identified (and predicted) *Cyp* gene family member. Many quantitative real-time polymerase chain reaction (qPCR) probe sets have now been empirically validated and are commercially available. This study utilizes qPCR to quantify multiple *Cyp*s and other eicosanoid-related gene changes in the rat hippocampus and parietal cortex at several time points after TBI. Determining the regulation of these genes and their eicosanoid products in specific brain regions and at specific time points after brain injury are the first steps in the investigation of their contribution to secondary brain damage and/or recovery of function.

## Results

### Custom low density array qPCR assays, normalization and negative controls

Parietal cortex and hippocampal cDNA from naïve and brain-injured or sham-operated rats at 6 h, 24 h, 72 h, or 168 h after surgery (n ≥ 3 per group) were used to determine the expression profiles of 96 genes in custom low density arrays by qPCR (see Additional file 1: Table S1 for probe set identifiers). The assays included 62 rat *Cyp* genes (37 known to metabolize ArA or other unsaturated fatty acids), plus 2 mouse *Cyp*s as negative controls; 16 eicosanoid-related genes that metabolize arachidonic acid or its primary metabolites; 8 eicosanoid receptor genes; as well as 5 inflammatory- or recovery-related genes; plus 3 highly expressed housekeeping genes.

Starting with equal quantities of total RNA, the housekeeping genes *Ppia* (peptidylprolyl isomerase A or cyclophilin-A, *CYC*); glyceraldehyde 3-phosphate dehydrogenase, and 18S rRNA were examined first to determine which to use for normalization purposes. *Ppia* was the least variant (Additional file 2: Figure S1), and subsequent qPCR C_t_ values for each target gene were normalized as the initial copy number ratio (ƒ_i_ that is proportional to 2^-ΔCt^, see Methods) to compare expression of the same gene between specimens.

Two cross-species negative controls were included in this study to test for amplification specificity in the highly conserved cytochrome P450 gene family. Probe sets for mouse *Cyp2c54* and *Cyp2c50* yielded no detectable amplification products in nearly all specimens. Unexpectedly, the probe set for mouse *Cyp2c54* showed expression in the injured hippocampus only at 24 h, in all 3 rats from this treatment group. Upon closer scrutiny, 5 distinct rat sequences were found to have homology within the probe set of mouse *Cyp2c54* (see Additional file 3). However, 3 of the rat genes examined in these analyses (*Cyp2c12, Cyp2c13, Cyp2c37*) showed no changes at any time point, and the 2 processed pseudogenes were not assayed. This result may characterize transient local expression of a pseudogene or a new rat *Cyp* homolog.

### Overview of gene expression changes

Forty-five of the 96 genes studied showed time-dependent changes in cortex or hippocampus after TBI (p < 0.05, Tukey HSD, Table 1). Sixteen arachidonate-metabolizing *Cyp*s (Table 1A) as well as 17 other eicosanoid-related genes (Table 1B) were temporally regulated in injured hippocampus and/or cortex. Moreover, several *Cyp*s with other known functions were regulated in the brain after TBI (Table 1C). Over half of the genes examined (51/96) showed differential expression levels between parietal cortex and hippocampus (p < 0.05, Tukey HSD, Additional file 1: Table S2). There were 12 genes showing sham effects; i.e., differences between naïve mRNA levels and shams at one or more time points (Additional file 1: Table S3). These were due to anesthesia or surgical intervention, and might have masked injury-related mRNA changes.

**Table 1:** **Changes in rat brain *cyp *and arachidonate-related gene expression after traumatic brain injury**

	**HIPPOCAMPUS, time postinjury:**				**CORTEX, time postinjury:**			
**Gene name**	**6 h**	**24 h**	**72 h**	**168 h**	**6 h**	**24 h**	**72 h**	**168 h**
	A. Arachidonate-related *Cyp*s: mRNA fold-changes*							
***Cyp2c6***	^b^	**3.5x**			^b^	^b^	^b^	^b^
***Cyp2c22***		**3x**						
***Cyp2c23***		**3x**						
***Cyp2c54***	^b^	**3x**	^b^	^b^	^b^	^b^	^b^	^b^
***Cyp2e1***		**2.5x**						
***Cyp2j3***				**2x**			**2x**	[1.5x]
***Cyp2j4***			[2x]	**4x**			**2x**^**¶**^	[1.5x]
***Cyp2u1***			**1.5x**	**2.5x**				
***Cyp4a1***		**3x**						
***Cyp4a8***	**2x**^**¶**^	**4x**^**¶**^	**7x**^**¶**^	**2x**^**¶**^				
***Cyp4b1***	[3x]	**6x**^**¶**^	**5x**	**9x**	**2x**^**¶**^	**3x**^**¶**^	**4x**^**¶**^	[1.5x]
***Cyp4f5***			**1.5x**	**2x**			[1.5x]	[1.5x]
***Cyp4f6***			**1.5x**	**2x**				
***Cyp4f18***					**3x**^**¶**^	[2x]	[1.5x]	
***Cyp4x1***					**½**x^**¶**^	**½**x	[**½**x]	**½**x ^**¶**^
***Cyp26b1***				**4x**^**¶**^				**2.5x**
	B. Eicosanoid-related genes: mRNA fold-changes*							
***Alox15***					**4x**	[2x]		
***Ptgds2***			**6x**	**8x**			**5x**^**¶**^	**4x**^**¶**^
***Ptges***	[2x]	[3x]	[3x]	**11x**	**14.5x**	[4.5x]	[1.5x]	[1.5x]
***Ptgis***			[1.5x]	**3.5x**	**2x**	**1.5x**^**¶**^	[2.5x]	[2.5x]
***Ptgs1***			**6x**	[½x]			**2.5x**	[½x]
***Ptgs2***	**3x**		[½x]	[½x]	**5x**	[3x]		
***Tbxas1***			**3x**	**5x**	[3.5x]	[3x]	**6x**^**¶**^	[3.5x]
***Ephx1***				**2x**				
***Hpgd***		**1.5x**						
***Lta4h***					[1.5x]		**2.5x**^**¶**^	[1.5x]
***Pla2g4a***	[2x]	[2x]	**3.5x**^**¶**^	**2.5x**^**¶**^	**2.5x**^**¶**^	**2.5x**^**¶**^	**5x**^**¶**^	[2x]
***Alox5ap***	[2x]	[3x]	**4x**	**4x**	**5x**	[3x]	[3x]	
***Ptgfrn***	**1.5x**	**1.5x**	**1.5x**	**1.5x**				
***Ptgdr_pred***			[1.5x]	**3x**			[2.5x]	[2.5x]
***Ptger2***	**2x**	[1.5x]	**3x**	[2x]	[2x]	[2.5x]	[2.5x]	
***Ptger4***	[2.5x]	[2.5x]	**5x**	**7x**	**2.5**^**¶**^	**1.5x**^**¶**^	**2.5x**^**¶**^	**1.5x**^**¶**^
***Ptgir***	6x^¶^	5x^¶^	3x^¶^	[2.5x]	**4x**	**2.5x**	[2x]	
	C. Other *Cyp *genes: mRNA fold-changes*							
***Cyp1a1***		**2.5x**^**¶**^	**1.5x**^**¶**^	**1.5x**^**¶**^				
***Cyp1b1***			**3x**	**20x**	**5x**^**¶**^	**6.5x**	**3.5x**^**¶**^	
***Cyp2a2***	^b^	[~10x]					^b^	
***Cyp2r1***			[1.5x]	2.5x				
***Cyp3a18***		**3x**						
***Cyp7a1***	^b^	4x^¶^	6.5x^¶^	8x^¶^		^b^	^b^	
***Cyp11b3***	9.5x^¶^	8.5x^¶^	2.5x^¶^					
***Cyp17a1***			**3x**					
***Cyp20a1***		[2x]	[2x]	**2.5x**				
***Cyp24a1***					~5.5x^¶^	~7x^¶^	^b^	
***Cyp27a1***	[½x]		[3x]	**9x**		[½x]	[1.5x]	[2x]
***Cyp27b1***				**2xª**	~8x^¶^	~11x^¶^	^b^	[~2x]

**A:** Arachidonate-related *Cyp*s; **B:** Eicosanoid-related genes; **C:** Other *Cyp* genes. Gene expression assessed by qPCR is expressed as fold-change “x” compared to sham-operated controls at the same time point (i.e., 2^-ΔΔCt^). Only changes greater than 1.5x (or less than ½x) are shown. *p < 0.05, 2-way ANOVA, Tukey HSD (except where noted). Analyses performed on 2^-ΔCt^ values of injured vs. sham at designated times after TBI (shown as fold-change rounded to the nearest ½x). ¶ p < 0.05, 1-way ANOVA with all shams combined as time zero. [x] brackets indicate changes ≥1.5-fold that were not statistically significant, but showed a trend (0.05 < p < 0.10). ^b^ Below the limit of quantification at these times. ~ Control (naïve, sham) values at or below limit of quantification, thus fold-change estimated based on highest control value(s).

### Temporal expression of eicosanoid-related expression after traumatic brain injury

Of the genes found to change over time, expression patterns of arachidonate-metabolizing and eicosanoid-related genes (Table 1A,B), as well as other *Cyp* genes (Table 1C) are presented below, organized by temporal pattern (i.e., time of onset and duration), with neuroanatomic differences noted for each.

Acute changes in mRNA levels were those initiated at 6 h or 24 h and did not persist at 72 h after TBI. In both hippocampus and parietal cortex, *Ptgs2* (cyclooxygenase-2, COX2) was increased at 6 h only. In hippocampus, acute-only increases included *Cyp2c6, Cyp2c22, Cyp2c23, Cyp2c54, Cyp2e1, Cyp4a1* and *Hpgd*. In cortex, acute-only increases included *Cyp4f18*, *Alox15*, *Alox5ap*, *Ptges*, *Ptgis* and *Ptgir*.

Early- and delayed-onset transient changes after TBI were defined as those changes lasting more than 24 h but not after 72 h postinjury. These included *Ptgs1* (72 h only) in both hippocampus and cortex; in hippocampus *Ptger2* (biphasic at 6 h, 72 h) and *Ptgir* (6 to 72 h); and in parietal cortex *Cyp4b1* (Additional file 4: Figure S2), *Pla2g4a* (6 to 72 h), *Cyp2j3* and *Tbxas1* (72 h only). It should be noted that changes detected at 72 h only might actually have been prolonged between 1d to 6d surrounding the 72 h timepoint.

Several mRNA levels changed acutely (within 24 h postinjury) and remained altered throughout the 7d observation period. These included *Cyp4b1* (Additional file 4: Figure S2), *Cyp4a8* and *Ptgfrn* in hippocampus; *Ptger4* and *Cyp4x1* (the only mRNA consistently decreased) in parietal cortex. Levels of *Lta4h* mRNA were increased biphasically, at 6 h and again at 72 h to 168 h in parietal cortex.

A number of genes showed delayed-onset prolonged changes (from 72 h to 168 h postinjury, or at 168 h only). In hippocampus *Alox5ap*, *Cyp2u1*, *Cyp4f5*, *Cyp4f6*, *Pla2g4a*, *Ptger4*, and *Tbxas1* were increased from 72 h to 168 h; whereas *Cyp2j3*, *Cyp2j4*, *Ptges*, *Ptgis*, *Ptgdr*, and *Ephx1* were increased at 168h only. In both hippocampus and parietal cortex, *Cyp26b1* and *Ptgds2* increased at 168 h only. It should be noted that changes detected at 168 h only might have occurred as early as 4d postinjury or only transiently around the 7d endpoint of this study.

### Temporal expression of steroid metabolizing and other *Cyp* genes after traumatic brain injury

Several other *Cyp* genes not known to metabolize ArA or polyunsaturated fatty acids showed altered levels after TBI (Table 1C). Acute only changes from 6 h to 24 h postinjury were seen in the cortex for *Cyp24a1* and *Cyp27b1*; and at 24 h only for *Cyp3a18* in hippocampus. Transient changes were seen for *Cyp11b3* and *Cyp17a1* in hippocampus, and for *Cyp1b1* in cortex. Acute-prolonged changes (6 h or 24 h through 168 h) were observed for *Cyp1a1* and *Cyp7a1* in hippocampus. Several were upregulated in a delayed-prolonged manner, including *Cyp1b1*, *Cyp2r1*, *Cyp20a1*, *Cyp27a1* and *Cyp27b1* in hippocampus. Interestingly, *Cyp2a2* mRNA appeared below detectable levels in the hippocampus in most sham and all naïve rats except at 24 h postinjury (suggesting gene induction), and 15/28 rats had below detectable levels in parietal cortex.

### Localization of *Cyp2j4* and *Cyp27a1* expression

**Figure 1:**
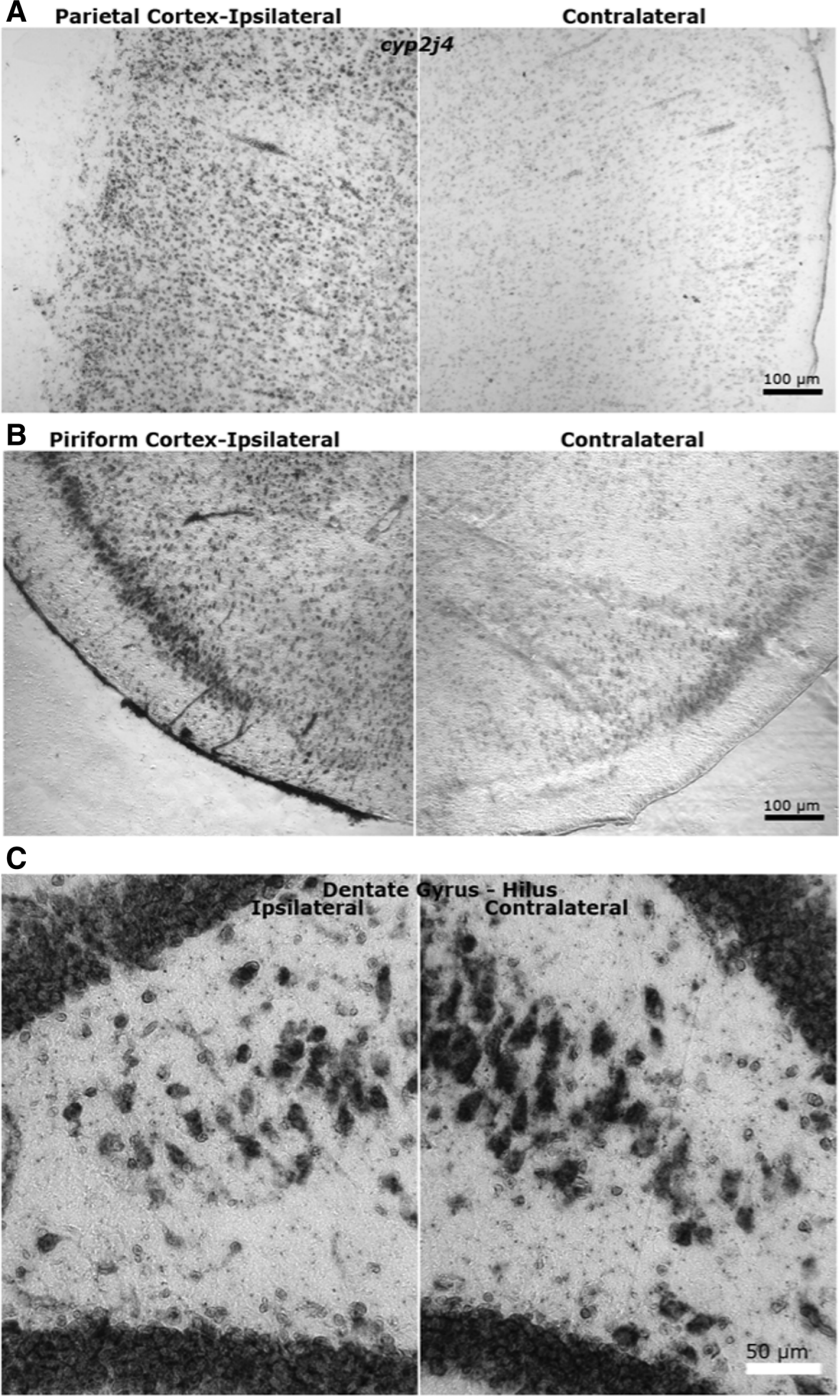
**In situ hybridization histochemistry for rat *Cyp2j4 *mRNA in brain 7 days after TBI.** Representative fields of injured and contralateral (**A**) parietal cortex; (**B**) piriform cortex; (**C**) hippocampal dentate gyrus. Note the apparent loss of pyramidal neurons in the ipsilateral dentate hilar region. Little staining was observed in the corpus callosum or other white matter structures. Some microvascular profiles appeared to stain positive in these non-perfused fresh frozen brain sections. Studies were carried out starting anterior to the site of injury, staining every sixth section through the injured volume (bregma −1.8 mm to approximately −4.5 mm, according to the coordinates of Paxinos and Watson [155]). High stringency hybridization and washes were performed as described in Section In situ hybridization histochemistry.

As a first step in localizing the expression of two brain injury regulated *Cyp*s of diverse function, in situ hybridization histochemistry was used to visualize *Cyp2j4* and *Cyp27a1* expression at 7d postinjury. Predominantly neuronal expression was seen for both *Cyp2j4* and *Cyp27a1* in neocortex. For *Cyp2j4* mRNA, cortical, hippocampal and habenular regions showed intense staining primarily in what appeared to be neuronal cells, with only light staining in the thalamus. Light staining of perivascular and other cell types was evident bilaterally. Little to no staining was observed in the corpus callosum or other white matter structures. Expression appeared greatest in injured parietal (Figure 1A) and piriform cortex (Figure 1B), compared with both sham and contralateral brain regions, whereas cingulate cortex showed expression bilaterally (not shown). Heaviest staining appeared in layer II cortical neurons, Ammon’s horn and dentate gyrus hippocampal neurons (Figure 1C), with many light to moderately stained cells in deeper cortical layers. Ipsilaterally, deep cortex and the hippocampal CA1 field showed a large number of lightly stained small cells (likely microglia or macrophages) that were not present contralaterally. In the midbrain, light staining was observed in some neuron-like and smaller cells, and in some microvascular and blood cells throughout the brain.

**Figure 2:**
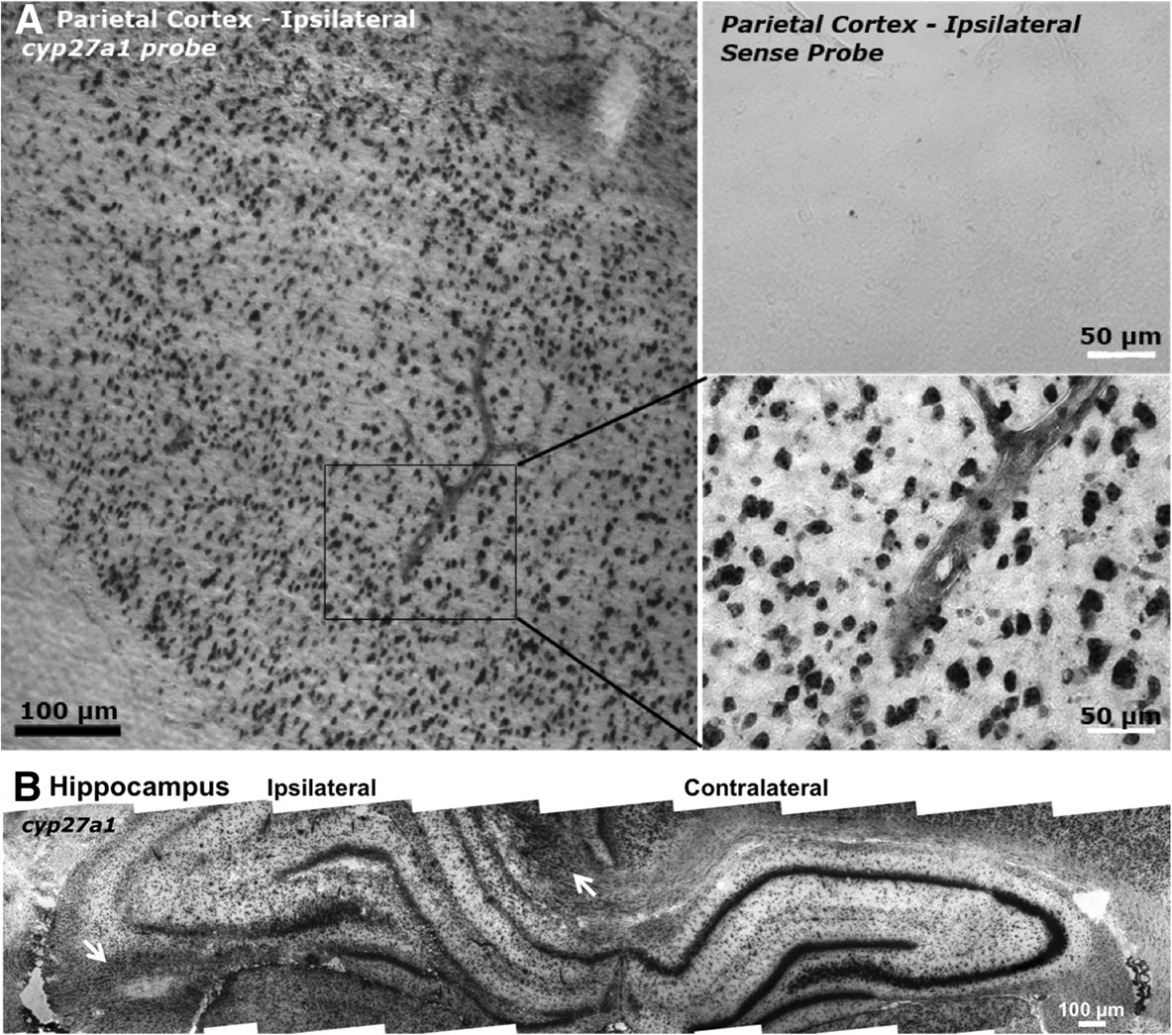
**In situ hybridization histochemistry for rat *Cyp27a1 *mRNA in brain 7 days after TBI.** (**A**) Representative fields of injured parietal cortex with higher magnification of negative control sense probe in the same region of a different section (top inset) and neuronal staining with a large vascular profile (bottom inset). (**B**) Composite showing injured and contralateral hippocampal *Cyp27a1* mRNA with ablation of neuronal staining, as well as heavier non-neuronal staining ipsilateral to injury. Some intense white matter staining is visible medially in the corpus callosum and the ipsilateral hippocampal fimbria (arrows). See Figure 1 legend for methodologic details.

A different pattern of staining was observed for *Cyp27a1* mRNA. Cingulate, parietal, perirhinal and piriform cortex showed *Cyp27a1* stained cells throughout all cortical layers. Intense staining was observed in many cortical layer II and deeper layer neurons (Figure 2A, B), as well as Ammon’s horn and dentate gyrus hippocampal neurons (Figure 2B). Medial habenula and some thalamic neurons were also intensely stained; and choroid plexus, third ventricle ependymal cells, and pial cells were moderately to intensely stained for *Cyp27a1* mRNA (not shown). Moderate stain was seen in some larger vascular profiles (but not microvasculature), as well as many (possibly oligodendroglial) cells in the corpus callosum, hippocampal fimbria, and other white matter structures (Figure 2B).

## Discussion

The parietal cortex and hippocampus are two brain regions that have been closely associated with the neurologic deficits observed in this model and in human brain injuries. Dynamic changes in mRNA levels were observed in these regions ipsilateral to TBI for many *Cyp* arachidonate monoxygenases and eicosanoid-related genes. A number of *Cyp* genes with other activities showed changes over time postinjury, as well. Altered mRNA levels do not unequivocally indicate altered enzymatic activities, however, nearly all changes showed higher mRNA levels, supporting their proposed function in the sustained surge of free fatty acid metabolism proximal to the site of injury during the hours and days after TBI. Altered eicosanoid metabolism at specific times after brain injuries contributes to dynamic local changes in the cerebral vasculature, inflammatory status, neural cell death, as well as neurophysiologic functions. It is not clear whether the observed responses to TBI are beneficial or not, but these results suggest brain region-specific changes in arachidonate metabolism due to alterations in the enzymatic machinery available at the time.

Histochemical detection of two *Cyp* mRNAs, one known to metabolize ArA (*Cyp2j4*) and the other known to metabolize cholesterol and vitamin D (*Cyp27a1*) showed different expression patterns at a time when both were induced in the injured brain. The distribution of *Cyp2j4* expression appeared to increase predominantly in cortical layer II neurons ipsilateral to injury, whereas *Cyp27a1* appeared more widespread in neurons and other cells throughout the brain. Staining for *Cyp27a1* was seen in many non-neuronal cells, particularly in lining cells of the brain and less intensely in white matter and larger vascular structures. Less non-neuronal staining was seen for *Cyp27a1*, in some smaller (likely glial) cells in deep cortex and hippocampus ipsitlateral to injury, as well as light staining in some microvascular structures.

The catalytic specificity of many *Cyp* monoxygenases show promiscuous substrate specificity (e.g., ArA, linoleic acid [56,59], etc.) and varying product profiles. This may have a number of implications after TBI, since CSF levels of many free fatty acids are elevated [38]. One Cyp enzyme may metabolize the same substrate differently in different tissues or under different conditions, thus, *Cyp* activities remain poorly defined. Still it may be useful to organize the observed changes in terms of the expected activities of the eicosanoid-related genes in the injured brain (assuming increased mRNA levels yield increases in active proteins).

### Functionality of temporally regulated genes

Early increases of prostanoid levels due to the rate-limiting enzyme COX2 in the injured brain, and their contribution to secondary injury has been well established [39,41-43,45,47,48,51,60-69]. This study revealed temporal regulation of prostanoid synthases, *Ptgs1* (COX1), *Ptgs2* (COX2), *Ptges* (PGE2 synthase, PGES), *Ptgds2* (PGD2 synthase-2), *Ptgis* (prostacyclin synthase), and *Tbxas1* (thromboxane A synthase 1) in hippocampus and cortex after TBI. In the cortex, *Ptgs2*, *Ptges* and *Ptgis* were acutely upregulated. Interestingly, *Ptgds2* and *Ptgs1* were elevated between 3 to 7d postinjury. Another picture emerged in the hippocampus. Whereas *Ptgs2* and *Ptgds2* were upregulated as in cortex, *Ptges* and *Ptgis* were upregulated much later in hippocampus, at 7d postinjury. The acute elevation in COX2 mRNA was consistent with previous results in the rat, where levels in hippocampus were greater than in cortex and COX2 mRNA returned to sham levels by 24 h postinjury [47,70]. *Ptges* is a prostaglandin (PG) synthase that catalyzes the formation of PGE2 from PGG2/PGH2 formed by cyclooxygenases. Its acute induction in the cortex was followed at 7d by an increase in hippocampus. The levels of PGE2 itself are elevated acutely after TBI in both the parietal cortex and hippocampus, and remain elevated for at least 3d postinjury [71], despite the increase in *Hpgd* mRNA (a prostaglandin dehydrogenase that converts PGs to their 15-keto metabolites) in hippocampus at 24 h postinjury. Contradictory to the finding of increased hippocampal *Ptges* at 7d, both tissue extraction [51,71] and hippocampal microdialysis studies [71] indicated a diminution of PGE2 levels at 6 to 7 days postinjury below that of sham animals. This increase may, therefore, represent a compensatory mechanism to return hippocampal PGE2 levels to steady state.

Thromboxane has been proposed to mediate hemostasis early after TBI by constricting blood vessels and promoting platelet activation and aggregation in damaged tissues [41,42,72,73]. Acute regulation of *Tbxas1* was not observed, either because it was transiently regulated before the initial 6 h time point, or possibly due to posttranscriptional regulation of this activity [74]. Unexpectedly, *Tbxas1* mRNA was elevated in ipsilateral hippocampus between 3 to 7d postinjury (with sustained non-significant rises in cortex). Except for *Tbxas1*, these enzymes have been associated with vasodilatory activities. The results suggest a dynamic balancing of cerebral vasodilatation and vasoconstriction proximal to the site of injury after TBI. Moreover, late expression of *Tbxas1* might have implications for astrocyte and microglial migration [75,76] or remyelination [77] in the injured brain.

Four of the eight prostanoid receptor genes examined had altered expression levels after TBI (Table 1B). The prostacyclin receptor *Ptgir* mRNA levels in both hippocampus and cortex appeared to rise acutely, in parallel with *Ptgis* in parietal cortex. In contrast, the PGD2 receptor *Ptgdr* mRNA was elevated at 7d in the hippocampus, while *Ptgds2* levels in both cortex and hippocampus were upregulated from 3 to 7d postinjury. In hippocampus, the prostaglandin receptor EP2 (*Ptger2*) was upregulated in what appeared to be a biphasic manner at 6 h and 3d postinjury. The prostaglandin receptor EP4 (*Ptger4*) was upregulated as well, from 3 to 7d in the hippocampus and throughout the time course in parietal cortex. Both these genes have been associated with the vasoactivity of PGE2, but also have been implicated in potential neuroprotective [78] and antiinflammatory [79,80] activities in the brain.

Early postinjury there was an upregulation in lipoxygenase-associated gene expression (*Alox15, Alox5ap*) in the cortex. Only *Alox5ap* mRNA was elevated in hippocampus, from 3 to 7d postinjury. Leukotriene products are known to promote neuroinflammation, increased vascular permeability, and edema [81-83]; but may also be associated with the production of potentially neuroprotective HETEs [84,85]. However, unlike formation of 12-HETE by *Cyp*s, the 12-lipoxygenase pathway produces potentially neurotoxic HPETE intermediates [86]. Perhaps in response, several *Cyp*s that metabolize leukotrienes (*Cyp4f18* at 6 h only, *Cyp4f5* and *Cyp4f6* at 3 to 7d, see below) as well as the leukotriene hydrolase *Lta4h* (at 3d postinjury) appeared to be upregulated in the cortex. Protein levels of *Lta4h* have only recently been characterized in the brain [87].

Of the sixty-five *Cyp* genes assayed, sixteen arachidonate-metabolizing *Cyp*s (Table 1A) and twelve other *Cyp*s (Table 1C) were regulated in the injured brain regions examined. In the hippocampus at 6 h after TBI, *Cyp2c6*, *Cyp2c22*, *Cyp2c23*, *Cyp2e1*, and *Cyp4a1* mRNAs were elevated (as was *Cyp2c54*, a mouse gene homolgous to rat *Cyp2c*’s, see Results). The increased levels of *Cyp2c22*, *Cyp2c23*, and perhaps *Cyp2c54* (homologous to human *Cyp2c8* and *Cyp2c9*) represent activities that synthesize various EETs [88-90], with predominantly vasodilatory activity [91-93]. Except for *Cyp4a1*, these enzymes have been associated with hypertension; namely, depletion of these activities appears to result in vasoconstriction and increased blood pressure [94-96].

An arachidonate ω-hydroxylase, *Cyp4a1* likely produces 20-HETE as its main product [97-99] (it also catalyzes ω-1 hydroxylation of fatty acids, ω-hydroxylation of prostaglandins [97]). In addition, hippocampal levels of *Cyp4a8*, also associated with 20-HETE formation [100], were elevated throughout the time course studied. Thus, *Cyp4a1* and *Cyp4a8* stand out as acute and chronic vasoconstrictive activities, respectively, particularly after brain injuries [7,9,100-103].

At later times postinjury, hippocampal *Cyp2j3*, *Cyp2j4*, *Cyp2u1*, *Cyp4f5*, and *Cyp4f6* levels were elevated. *Cyp2j3* and *Cyp2j4* likely produce mainly EETs [52,92,104]. *Cyp2u1* catalyzes predominantly 19- and 20-HETE formation [105], whereas the *Cyp4f5* and *Cyp4f6* hydroxylases appear to inactivate leukotrienes [50,106,107]. As with the prostanoid synthases, it appears that regulation of both vasodilatory and vasoconstrictive *Cyp* activities may be required to balance the circulatory needs of the injured hippocampus.

In the cortex, only two *Cyp* arachidonate monoxygenases were acutely upregulated after TBI. At 6 h, *Cyp4f18*, and from 6 h to 3d postinjury *Cyp4b1* mRNA levels were elevated. Both these genes have been associated with potentially neuroprotective activities. *Cyp4f18* metabolizes proinflammatory leukotrienes [108] and *Cyp4b1* has been implicated in 12-HETE synthesis in ocular tissues [84,85,109]. Hampson and Grimaldi [28] found that 12-HETE can protect neurons from glutamate-mediated excitotoxicity in vitro. Interestingly, while elevated *Cyp4b1* mRNA was seen at 6 h in the cortex, its elevation was delayed until 24 h postinjury in the hippocampus, a site of extensive apoptotic cell death [110-112].

Another conspicuous finding was the reduced mRNA level of *Cyp4x1* in the cortex throughout the postinjury period. This gene is predominantly expressed in brain [113] and has recently been characterized in converting the endocannabinoid anandamide to a single monooxygenated product, 14,15-epoxyeicosatrienoic ethanolamide [114,115]. The observed reduction of this activity might contribute to endocannabinoid-mediated neuroprotection after TBI [116,117].

Several *Cyp*s not directly associated with polyunsaturated fatty acid metabolism also showed time- and tissue-dependent changes after TBI that might be relevant to secondary injury and recovery. *Cyp27a1*, the sterol 27-hydroylase, was differentially expressed after TBI. Hippocampal *Cyp27a1* mRNA levels increased 3- to 8-fold at 72 h to 168 h postinjury, respectively, whereas cortical levels did not appear to change. Histochemistry revealed cells intensely stained for *Cyp27a1* mRNA throughout the cortex, deep brain structures, and meninges, choroid plexus and ependymal cells. Peripherally, this enzyme metabolizes bile acids, cholesterol and vitamin D3, and has been suggested to contribute to neurodegenerative disease by increasing cholesterol penetration across the blood brain barrier by conversion to 27-hydroxycholesterol [118]. Conversely, intrinsic brain expression of *Cyp27a1* might enhance the egress of cholesterol, released from damaged cell membranes, from the brain to the peripheral circulation.

Two of the *Cyp*s acutely induced in the brain after TBI have been shown to be regulated by acute stress-related stimuli (e.g., stress hormones, ethanol toxicity). For example, *Cyp2c6* is rapidly induced by glucocorticoids in rat hepatoma cells [119], and is a close homolog of human *Cyp2c8* and *Cyp2c9* implicated in eicosanoid production [120-122]. Also acutely regulated, *Cyp2e1* is a major ethanol-inducible gene in the liver and metabolizes arachidonic acid to HETEs [123]. Its expression is ~1000-fold lower in the hippocampus than the liver, and is induced in neural cells by high concentrations of ethanol [124].

Surprisingly, *Cyp2e1* inhibition has been shown to increase rat brain dopamine levels, implicating it in catecholamine biotransformation [125]. Selected members of the *Cyp2d* subfamily (*Cyp2d2, 2d4* or *2d18*) also have been shown to synthesize dopamine in vitro and in vivo [126,127], although none of these genes were regulated in hippocampus or cortex after TBI. Dopamine levels decrease acutely in the traumatically injured rat cortex, but showed increased levels in hypothalamus and striatum as soon as 1h or 6h postinjury, respectively [128]. These regional changes might reflect regional decreases in *Cyp2e1* expression, or increased *Cyp2d* activity. Further study of hypothalamic and striatal gene expression may clarify the contribution of *Cyp*s to acute dopamine fluxes after TBI.

In addition to its possible role in EET production, *Cyp2c22* is both regulated by and able to metabolize all-*trans* retinoic acid [90]. Its acute regulation after TBI suggests the possibility of transducing cross-lipid signalling, perhaps with respect to neuroinflammatory cell proliferation [90]. In addition, *Cyp26b1* metabolizes retinoic acid, including “all trans” retinoic acid, a process thought to contribute to regulation of spatial patterning during neurogenesis [129-131] and neuroplasticity [132,133]. Induced in both cortex and hippocampus at 7d postinjury, this enzyme might contribute to neuroplasticity in the injured brain, possibly in the formation of neuronal-astroglial boundaries.

TBI-mediated regulation of *Cyp17a1* was observed only in the hippocampus, being elevated at the 24 h time point. *Cyp17a1*, the steroid 17alpha-hydroxylase/17,20-lyase, is one of the key enzymes in glucocorticoid, dehydroepiandrosterone, and androgen biosynthesis [134]. Its activity in the brain, until fairly recently a matter of debate, has been established [135-137]. This activity is known to metabolize progesterone, a neuroprotective neurosteroid [138-140] now in phase III clinical trials for treatment of TBI [141,142]. This is also a critical step in the biosynthesis of other potentially neuroprotective neurosteroids, including dehydroepiandrosterone [70,143,144], testosterone [145,146], and estradiol [147-150]. It remains to be determined whether the enhancement of this activity (or the others described above) after brain injury would improve the recovery phenotype.

Tissue-specific differences between cortex and hippocampus were observed in basal mRNA levels in over half the genes examined. In addition, hippocampus and cortex responded differently in most cases of injury-induced changes, as well. Nonetheless, nine genes were coordinately regulated, increasing approximately the same extent, at similar time points in both tissues. These were *Alox5ap*, *Cyp2j4*, *Cyp26b1*, *Pla2g4a*, *Ptgir*, *Ptgs1*, *Ptgs2*, *Ptgds*, and *Tbxas1*. This might be attributable to release of factors into the blood or CSF, or neurotransmitter-mediated induction of these genes in multiple brain regions. Global changes were also observed in comparing naïve and sham-operated animals in a number of genes (Additional file 1: Table S3), suggesting that anesthesia and/or craniotomy induced global, sometimes lasting changes in the brain.

## Conclusion

This qPCR study has established detectable mRNA levels for several *Cyp*s not previously described in the brain (see Additional file 1: Table S1 for genes detected in cortex and hippocampus). This suggests the presence of these activities, despite any lack of change postinjury. There will be, going forward, a need for further study to better understand these results. For example, only changes greater than 50% in parietal cortex or hippocampus were analyzed in this study due to the large number of genes examined. Based on these mRNA and limited histochemical results, it would be of interest to determine the regulation of these *Cyp* and eicosanoid-related genes in more distal brain regions, e.g., hypothalamus, dorsolateral thalamus, piriform cortex or amygdala, where neurologic dysfunction might also impact brain injury recovery. These and more subtle changes in gene expression, and in other brain regions, likely contribute to the overall maintenance of lipid metabolism in the healthy brain, and have implications for neural changes in arachidonate metabolism in response to injury.

The dynamic environment in the brain after traumatic injury likely requires different metabolic events at different times for optimal repair to proceed. It may be speculated that timely regulation of arachidonic acid metabolism contributes to functional recovery after TBI. To determine whether these eicosanoid-related changes in gene expression are adaptive or pathologic, pharmacologic and/or genetic manipulation of each gene (product) will need to be performed *at a given time after brain injury*. Thus, the investigation of arachidonic acid metabolism in the brain continues to be a fertile field of investigation.

## Methods

### Traumatic brain injury

All animal protocols used were approved by the University of Cincinnati IACUC. The lateral cortical contusion rat TBI model was carried out as previously described [47] with minor modifications. Sprague–Dawley rats, (male, 300-400 g Harlan) were pre-anaesthetised with isoflurane and maintained at a surgical level of anaesthesia with a facemask using oxygen and isoflurane. After immobilisation in a stereotaxic device, a 6 mm craniotomy was performed over the parietal cortex between the left lateral ridge and the sagittal suture, midway between lambda and bregma. Animals were randomly assigned to sham or injury groups. Traumatic brain injury was induced via a cortical contusion using a pneumatic piston (5 mm diameter, 4 m/s, 100 ms) to a depth of 2.7 mm. Sham operated controls were surgically prepared but not injured. Postsurgical neurological assessments were performed just after removing aneasthesia for exclusion criteria.

### Sample preparation

Conscious animals were decapitated using a sharpened guillotine at preselected times of 6, 24, 72 and 168 hours (n = 4 per injured time point, n = 3 per sham time point, n = 4 naïves). Brain regions were rapidly dissected on ice and flash frozen on powdered dry ice. Tissue samples were stored at −80°C until RNA extraction. Ipsilateral parietal cortex, ~100 mg, not including the site of injury was used; for hippocampus, the entire ipsilateral structure was used. Total RNA was isolated by sonicating frozen tissue in TRI reagent (Molecular Research Labs, Cincinnati, OH), following the protocol through chloroform extraction. Aqueous phase was dissolved in RNA lysis buffer (RNeasy kit, Qiagen, Chatsworth, CA), and RNA purified according to kit protocol, including on-column DNase. RNA quality was assessed by spectrometry, microfluidics and denaturing gel electrophoresis. All sample A_260/280 nm_ ratios were 1.85 – 2.10, RNA integrity numbers of 6.5 ± 0.5 were obtained (Total RNA Nano Series II, Agilent), and ribosomal RNAs comprised 21.2 ± 3.8% of total RNA (mean ± SD of all samples).

A high capacity cDNA reverse transcription kit (random hexamer primers, ABI) was used to generate cDNA from total RNA (2 µg, 2 h, 37°C); duplicate reactions provided enough template for all assays. Aliquots of each sample cDNA were stored at −80°C.

### Real-time PCR

Quantitative real-time polymerase chain reaction (qPCR) studies were carried out in conformity with recommended guidelines [151]. Sample cDNAs were combined with 2× Universal PCR Master Mix (ABI, Applied Biosystems, Inc., Foster City, CA) in a custom 96 well plate array (ABI, see Additional file 1 for details) using an ABI PRISM 7500 system (courtesy of Allyson Cole Strauss in the lab of Prof. Jack Lipton, U. Cincinnati Department of Psychiatry). Each TaqMan® Gene Expression Assay (ABI) consisted of two sequence-specific PCR primers and a TaqMan assay-FAM™ dye-labeled MGB probe. A quantity of 60 ng cDNA in a 20 µL reaction was chosen because in cortex, 96% of the probe sets (in hippocampus, 84%) fell within the dynamic range after 40 cycles of PCR.

Initially, 8 cDNA samples were evaluated in triplicate and the interassay variance was found to be very small (coefficients of variation less than 3% for all assays). Thereafter, 3 – 4 biological replicates were assayed using one technical replicate. Acrylamide gel analyses of these reactions showed appropriately sized amplicon bands throughout, and “no reverse transcriptase” controls showed no bands. Assays with very low abundance targets C_t_ > 37.4 (4% of cortex samples, 15% of hipocampal samples) were judged not abundant enough for precise quantification and were assigned an expression value of zero.

Consistent reaction efficiencies were confirmed for relative quantification using a reference gene [152]. Serial dilutions of the initial 8 sample cDNAs were assayed on the custom plate arrays. When C_t_ was graphed against log cDNA input for each assay and dilution, the mean slope was −2.80 ± 0.04 (ideal slope = −3.32 [152]), indicating low variability and high amplification efficiencies throughout the assays. Efficiencies of very low abundance genes (undiluted C_t_ ≥ 35.9) could not be evaluated by this method, however, because serial dilution brought C_t_ values below the range of detection. Further validation was carried out by graphing ΔC_t_ versus log cDNA input [153], yielding absolute value slopes less than 0.1 (0.08 ± 0.02, excluding the very low abundance assays).

### Data analysis

Results from qPCR were calculated from C_t_ values (the estimated number of PCR cycles to reach the threshold fluorophore release, when signal > 10 standard deviations of fluoresence background, indicating entry into the exponential phase of amplification, ABI 7500 manual) generated for each probe set. Gene expression is most often represented as a normalized value, i.e., with respect to an invariant housekeeping gene, sample protein, or tissue weight. For profiling the large number of genes in this study, we used a normalized representation of the target (*i*) to reference (*ref*) gene copy number ratio based on the exponential of ΔC_t_ = (C_t*i*_ - C_t*ref*_) [153,154]. The number of cycles, C_t_ is related to the initial copy number of cDNA (N_0_) in the sample by the equation N_0*i*_ = k_*i*_ (1 + E_*i*_)^-Ct*i*^, where k_*i*_ is a constant that directly relates N_0*i*_ to copy number, and E_*i*_ is the efficiency of the PCR amplification, ranging between 0 and 1. Dividing N_0*i*_/N_0*ref*_ yields a constant, K_*i*_ = k_*i*_/k_*ref*_, that depends on the individual properties of each probe set [153]. When the target efficiency and reference efficiency are comparable (within about 10% of each other [154], see above) they can be assumed to be equal, and the initial copy number ratio ƒ_i_ = N_0*i*_/N_0*ref*_ = K_*i*_ 2^-ΔCt^. Since K_*i*_ is a constant for each probe set, 2^-ΔCt^ is directly proportional to the ratio of target to reference initial copy number for each sample and assay, and can be treated as a continuous variable, amenable to standard statistical approaches [152,154]. The 2^-ΔCt^ values were evaluated by two-way ANOVA (independent variables: treatment, time) with Tukey HSD post-hoc tests. A separate one-way ANOVA was performed with all shams combined. A p-value less than 0.05 was required to reject the null hypothesis that group means were equivalent. For presentation purposes in Table 1 (n-fold mRNA levels), the mean 2^-ΔCt^ values from injured tissue were normalized to shams at the same time point.

### In situ hybridization histochemistry

Cellular localization of eicosanoid-related gene expression was carried out on 20 µm sections of fresh frozen brain tissue by in situ hybridization histochemistry utilizing internally labeled complementary RNA probes. Sections from injured and sham-operated rat brains (n = 3 each, 7d postinjury) were chosen starting anterior to the site of injury and then every sixth section through the injured volume (Bregma −1.8 mm to approximately −4.5 mm, according to the coordinates of Paxinos and Watson [155]). Sequences unique for the individual *Cyp* genes (as determined by BLAST analyses) were derived from the 3’-end of the coding region and a portion of the untranslated region, spanning a putative exon-exon splice junction. These sequences were amplified by high fidelity PCR (Roche Applied Science, Penzberg, Germany) from rat brain cDNA and cloned into pGEM (Promega, Madison, WI) vectors. For rat *Cyp2j4*, the insert included nucleotides 1188 to 1585 (NM_023025); for rat *Cyp27a1*, the insert included nucleotides 1361 to 1766 (NM_178847), both with respect to the translation initiation start site. Plasmids were linearized to provide a template for RNA synthesis directed by a viral T7 or SP6 promoter to produce either antisense or sense (negative control) probes. RNA was synthesized according the the manufacturer’s instructions (Roche digoxigenin labeling kit), template DNA degraded (RQ1 ribonuclease-free DNase, Promega) and RNA probes purified on spun columns (Qiagen, Chatsworth, CA). All probes were size analyzed on denaturing 6% polyacrylamide-urea gels. Probe concentration and quality were assessed by absorbance at 260 nm (A_260_) and the A_260_/A_280_ ratio. Studies for each probe (and controls) were carried out in parallel with equal mass of probe, hybridization and development times. Prehybridization and hybridization were carried out in 50% formamide, essentially as described [156], with 400 ng/mL RNA probes under high stringency conditions to minimize cross-reactivity between the target genes and closely related *Cyp* family members. Overnight incubation was at 50°C in a humidified chamber. All washes were 15 min at 50°C: twice in 2xSSC (SSC is 150 mM sodium chloride, 15 mM sodium citrate, pH 7.4), twice in 1xSSC, twice in 0.1xSSC. Staining was performed exactly as described (DIG Wash & Block Buffer Set, DIG Staining Solution, Roche) by incubating sections with wash buffer, blocking reagent, then alkaline phosphatase-conjugated anti-digoxigenin F_ab_ fragments (1:250, 1 h in a humidified chamber at room temperature), washing twice and developing the color overnight at room temperature in the presence of 1 mM levamisole (Sigma, St. Louis, MO) to inhibit endogenous phosphatase activity. Sections were washed (50 mM tris, 5 mM EDTA, pH7.4), rinsed in distilled water, dehydrated in increasing ethanol washes, and coverslipped with Permount (Fisher Scientific) for observation under bright field microscopy.

## Competing interests

The authors have no competing interests.

## Authors’ contributions

KIS designed the study, performed the statistical analyses and drafted the manuscript. RC participated in the design of the study and determined the sequence specificity of the probes and assisted in the sequence selections. MB and RM determined the specificity of the ABI probe sets, carried out the qPCR genomic studies, and participated in molecular cloning. All authors approved the final manuscript.

## Supplementary Material

Additional file 1: Table S1 Detection of mRNA in naïve, sham, and injured hippocampus (HCI) or cortex (CXI). Positive or negative qPCR assay detection is shown for each gene, along with the ABI assay identification and a description of the gene. The 2^-ΔCt^ for each of the assayed genes is shown in Tables S4 and S5. **Table S2.** Regional differences in mRNA levels in naïve and sham brains. Combined data from naïves and shams are compared to show distinct levels of mRNA expression between ipsilateral parietal cortex and hippocampus. **Table S3.** Sham effects on mRNA levels in naïve and sham brains. Comparison of gene expression in naïves and shams at different times postsurgery showed a number of sham effects, evaluated by 1-way ANOVA with naïves at time 0, and shams at 6 h, 24 h, 72 h, and 168 h. p < 0.05, ANOVA, Tukey HSD, except where bracketed, 0.05 < p < 0.10. **Table S4.** Mean and standard error expression values for ispilateral hippocampus (n = 3–4 for each). **Table S5.** Mean and standard error expression values for ispilateral parietal cortex (n = 3–4 for each). Click here for file

Additional file 2: Figure S1 Graph of C_t_ values from three “housekeeping” genes assayed by qPCR in parietal cortex. A total of 29 animals were assessed, with n = 4 naïves (time 0), n = 3 shams per time point, and n = 4 injured per time point. One *Gapdh* point (C_t_ = 39.4, 72 h sham) was removed as an outlier. The least variant of these genes over the entire data set was *Ppia* (cyclophilin A), that was used for normalization of all subsequent data. Click here for file

Additional file 3 **Cross species control sequence comparisons between mouse and rat *Cyp*s.** Click here for file

Additional file 4: Figure S2 Induction of *Cyp4b1* mRNA in injured parietal cortex and hippocampus after brain injury. Acutely after TBI, *Cyp4b1* was elevated in ipsilateral parietal cortex (CXI) and remained elevated for at least 3d postinjury compared to shams. In ipsilateral hippocampus (HCI), several-fold elevations occurred starting 24 h and continued for at least 7d postinjury. Dotted lines represent mean naïve mRNA levels (n = 4); n = 3 shams and n = 4 injured per time point. *p < 0.05, 2-way ANOVA, Tukey HSD vs. shams at same time point. ¶p < 0.05, 1-way ANOVA, Tukey HSD, with shams combined at time zero. Click here for file
