# Supplementary figures and images for: Temporal changes of cytochrome P450 (Cyp) and eicosanoid-related gene expression in the rat brain after traumatic brain injury
Source: BMC Genomics. 2013 May 4;14:303. doi: 10.1186/1471-2164-14-303 (PMC3658912; doi:10.1186/1471-2164-14-303)

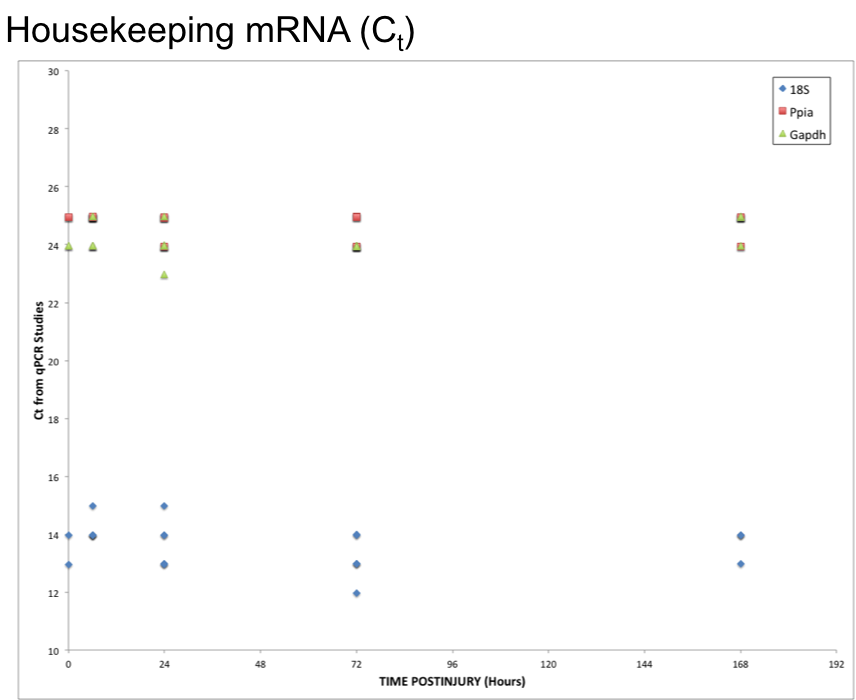

Supplement: Additional file 2: Figure S1 — Graph of Ct values from three “housekeeping” genes assayed by qPCR in parietal cortex. A total of 29 animals were assessed, with n = 4 naïves (time 0), n = 3 shams per time point, and n = 4 injured per time point. One Gapdh point (Ct = 39.4, 72 h sham) was removed as an outlier. The least variant of these genes over the entire data set was Ppia (cyclophilin A), that was used for normalization of all subsequent data. [file 1471-2164-14-303-S2.tiff]

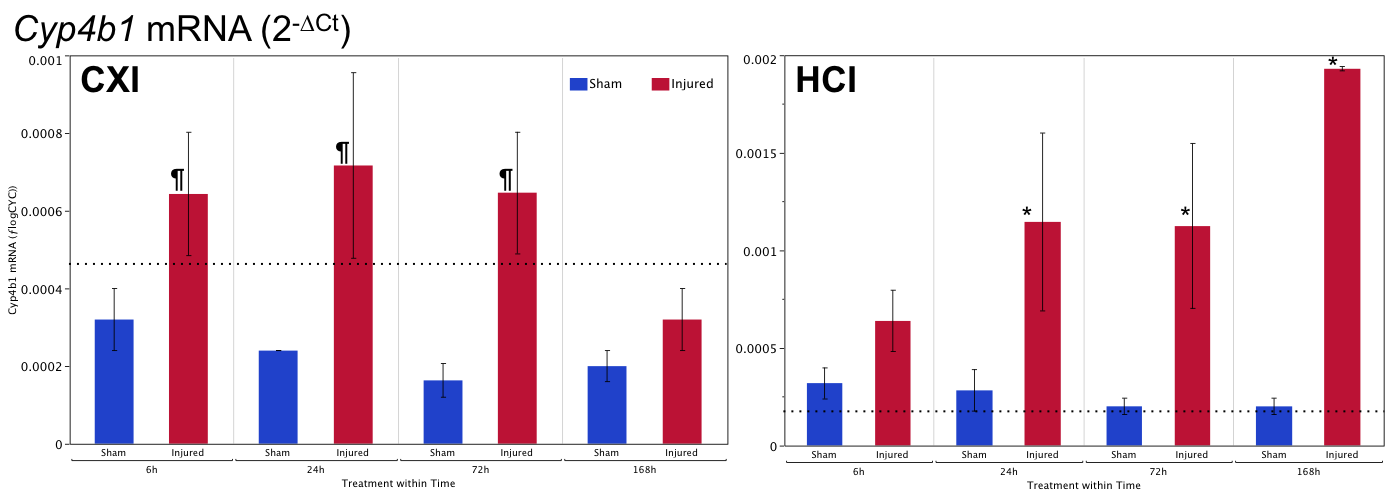

Supplement: Additional file 4: Figure S2 — Induction of Cyp4b1 mRNA in injured parietal cortex and hippocampus after brain injury. Acutely after TBI, Cyp4b1 was elevated in ipsilateral parietal cortex (CXI) and remained elevated for at least 3d postinjury compared to shams. In ipsilateral hippocampus (HCI), several-fold elevations occurred starting 24 h and continued for at least 7d postinjury. Dotted lines represent mean naïve mRNA levels (n = 4); n = 3 shams and n = 4 injured per time point. *p < 0.05, 2-way ANOVA, Tukey HSD vs. shams at same time point. ¶p < 0.05, 1-way ANOVA, Tukey HSD, with shams combined at time zero. [file 1471-2164-14-303-S4.tiff]
