# Supplementary material for: Temporal changes of cytochrome P450 (Cyp) and eicosanoid-related gene expression in the rat brain after traumatic brain injury
Source: BMC Genomics. 2013 May 4;14:303. doi: 10.1186/1471-2164-14-303 (PMC3658912; doi:10.1186/1471-2164-14-303)
Supplement: Additional file 3 — Cross species control sequence comparisons between mouse and rat Cyps. [file 1471-2164-14-303-S3.docx]

Supplemental Data

Two cross-species negative controls were included in the study to test for amplification specificity in this highly conserved gene family. The mouse cyp2c54 and cyp2c50 probe sets yielded no detectable amplification in nearly all cDNA specimens examined. However, mouse cyp2c54 showed elevated levels in all 3 rat cDNAs from the hippocampus at 24 h postinjury (but not in 24 h shams). This result was replicated, and the amplicon was characterized by nondenaturing acrylamide gel electrophoresis to be approximately 125 bp (expected size of 109 bp). Comparisons of mouse cyp2c54 probe regions (ABI will not disseminate exact probe sequences) spanning exons 5 and 6 for similarities to rat mRNAs yielded several potential homologies. The BLAST analysis yielded 41 rat sequences (of 250 results) homologous to the probe region of mouse cyp2c54. Of these, 19 records had matches of between 16 to 23 base pairs in both flanking regions that could yield amplicons of 138 to 145 bp. These 19 records represented 3 distinct genes and 2 processed psuedogenes (-ps). These comprised, in order of greatest homology, cyp2c13, cyp2c77-ps, cyp2c37 variant 2 (PB1-2, as well as PB1-1), cyp45R-ps, and cyp2c12. Of these, only the pseudogenes were not included in the study, thus it is likely this aberrant result represents expression of a processed pseudogene at 24 h postinjury.

ABI probe set Mm02602271_mH yields an amplicon of 109 bp and spans the exon 5|6 junction. Bases 711 to 920 of the mouse cyp2c54 coding region comprised over 105 bp on either side of this exon-exon junction, and must include the sequences in the ABI probe set. This sequence was used for a BLAST comparison (blastn, 250 results returned). The rat sequences returned that had homology in flanking regions enough to yield an amplicon of similar size are reported below. Underlines indicate potential priming sites, while sites that have mismatches compatible with primer binding are marked in strikethrough.

BLAST Analysis for Sequence: Mus Cyp2c54

Search from 711 to 920 Program: blastn

Expect: 1e+02 Low complexity filter: on Matrix: n/a

Genetic Code: n/a Gapped search: on

Open cost: 5 Extend cost: 2

Database: nr (37,008,494,498 residues in 16,156,712 sequences)

Karlin-Altschul Statistics: Kappa = 0.41, Lambda = 0.625, Entropy = 0.78

19. gi|298904201|emb|FQ218917.1| Rattus norvegicus TL0ABA49YB24 mRNA sequence

Length = 1121

Score = 176.2 bits (194), Expect = 5.8e-41

Identities = 160/202 (79%), Positives = 160/202 (79%), Gaps = 0/202 (0%)

Query: 719 TTAAAAATTTCCTTTTGGAGAAAATAAGGGAACATAAAGAATCATTGGATGTTACAATTCCTCGGGACTTTATTGATTATTTCCTAATTAAAGGAGCCCA

|||| | || ||||||||||||||||| |||||| ||||||| ||||||||| ||| |||||||||||| ||||||||||| |||||| || || ||

Subject: 100 TTAAGAGTTACCTTTTGGAGAAAATAAAAGAACATGAAGAATCGTTGGATGTTTCAAATCCTCGGGACTTCATTGATTATTTTCTAATTGAAAGAAATCA

Query: 819 GGAAGATGACAATCATCCTTTGAAAAATAATTTTGAACACCTGGCAATAACAGTGACTGATCTTTTTATTGGTGGGACAGAGTCAATGAGCACAACACTG AG

|||| ||| |||||| |||| ||| |||||||||||||||| | ||||||||| | ||| ||| ||| | |||| || | ||| ||||| || ||

Subject: 200 GGAAAATGCCAATCAGTGGATGAACTATACACTTGAACACCTGGCAATCATGGTGACTGATTTGTTTTTTGCTGGAATAGAGACAGTAAGCTCAACAATG AG

20. gi|56972349|gb|BC088105.1| Rattus norvegicus cytochrome P450 2c13, mRNA (cDNAclone MGC:108565 IMAGE:7372890), complete cds

Length = 1768

Score = 176.2 bits (194), Expect = 5.8e-41

Identities = 160/202 (79%), Positives = 160/202 (79%), Gaps = 0/202 (0%)

Query: 719 TTAAAAATTTCCTTTTGGAGAAAATAAGGGAACATAAAGAATCATTGGATGTTACAATTCCTCGGGACTTTATTGATTATTTCCTAATTAAAGGAGCCCA

|||| | || ||||||||||||||||| |||||| ||||||| ||||||||| ||| |||||||||||| ||||||||||| |||||| || || ||

Subject: 752 TTAAGAGTTACCTTTTGGAGAAAATAAAAGAACATGAAGAATCGTTGGATGTTTCAAATCCTCGGGACTTCATTGATTATTTTCTAATTGAAAGAAATCA

Query: 819 GGAAGATGACAATCATCCTTTGAAAAATAATTTTGAACACCTGGCAATAACAGTGACTGATCTTTTTATTGGTGGGACAGAGTCAATGAGCACAACACTG AG

|||| ||| |||||| |||| ||| |||||||||||||||| | ||||||||| | ||| ||| ||| | |||| || | ||| ||||| || ||

Subject: 852 GGAAAATGCCAATCAGTGGATGAACTATACACTTGAACACCTGGCAATCATGGTGACTGATTTGTTTTTTGCTGGAATAGAGACAGTAAGCTCAACAATG AG

21. gi|71051772|gb|BC098903.1| Rattus norvegicus cytochrome P450 2c13, mRNA (cDNAclone MGC:114253 IMAGE:7373446), complete cds

Length = 1757

Score = 176.2 bits (194), Expect = 5.8e-41

Identities = 160/202 (79%), Positives = 160/202 (79%), Gaps = 0/202 (0%)

Query: 719 TTAAAAATTTCCTTTTGGAGAAAATAAGGGAACATAAAGAATCATTGGATGTTACAATTCCTCGGGACTTTATTGATTATTTCCTAATTAAAGGAGCCCA

|||| | || ||||||||||||||||| |||||| ||||||| ||||||||| ||| |||||||||||| ||||||||||| |||||| || || ||

Subject: 731 TTAAGAGTTACCTTTTGGAGAAAATAAAAGAACATGAAGAATCGTTGGATGTTTCAAATCCTCGGGACTTCATTGATTATTTTCTAATTGAAAGAAATCA

Query: 819 GGAAGATGACAATCATCCTTTGAAAAATAATTTTGAACACCTGGCAATAACAGTGACTGATCTTTTTATTGGTGGGACAGAGTCAATGAGCACAACACTG AG

|||| ||| |||||| |||| ||| |||||||||||||||| | ||||||||| | ||| ||| ||| | |||| || | ||| ||||| || ||

Subject: 831 GGAAAATGCCAATCAGTGGATGAACTATACACTTGAACACCTGGCAATCATGGTGACTGATTTGTTTTTTGCTGGAATAGAGACAGTAAGCTCAACAATG AG

22. gi|203869|gb|M33994.1|RATCYPMS Rat male-specific liver cytochrome P-450 g mRNA, complete cds

Length = 1720

Score = 176.2 bits (194), Expect = 5.8e-41

Identities = 160/202 (79%), Positives = 160/202 (79%), Gaps = 0/202 (0%)

Query: 719 TTAAAAATTTCCTTTTGGAGAAAATAAGGGAACATAAAGAATCATTGGATGTTACAATTCCTCGGGACTTTATTGATTATTTCCTAATTAAAGGAGCCCA

|||| | || ||||||||||||||||| |||||| ||||||| ||||||||| ||| |||||||||||| ||||||||||| |||||| || || ||

Subject: 719 TTAAGAGTTACCTTTTGGAGAAAATAAAAGAACATGAAGAATCGTTGGATGTTTCAAATCCTCGGGACTTCATTGATTATTTTCTAATTGAAAGAAATCA

Query: 819 GGAAGATGACAATCATCCTTTGAAAAATAATTTTGAACACCTGGCAATAACAGTGACTGATCTTTTTATTGGTGGGACAGAGTCAATGAGCACAACACTG AG

|||| ||| |||||| |||| ||| |||||||||||||||| | ||||||||| | ||| ||| ||| | |||| || | ||| ||||| || ||

Subject: 819 GGAAAATGCCAATCAGTGGATGAACTATACACTTGAACACCTGGCAATCATGGTGACTGATTTGTTTTTTGCTGGAATAGAGACAGTAAGCTCAACAATG AG

23. gi|25453405|ref|NM_138514.1| Rattus norvegicus cytochrome P450, family 2, subfamily c, polypeptide 13 (Cyp2c13), mRNA

>gi|203769|gb|M32277.1|RATCYP45GA Rat cytochrome P-450g mRNA, complete cds

Length = 1721

Score = 176.2 bits (194), Expect = 5.8e-41

Identities = 160/202 (79%), Positives = 160/202 (79%), Gaps = 0/202 (0%)

Query: 719 TTAAAAATTTCCTTTTGGAGAAAATAAGGGAACATAAAGAATCATTGGATGTTACAATTCCTCGGGACTTTATTGATTATTTCCTAATTAAAGGAGCCCA

|||| | || ||||||||||||||||| |||||| ||||||| ||||||||| ||| |||||||||||| ||||||||||| |||||| || || ||

Subject: 730 TTAAGAGTTACCTTTTGGAGAAAATAAAAGAACATGAAGAATCGTTGGATGTTTCAAATCCTCGGGACTTCATTGATTATTTTCTAATTGAAAGAAATCA

Query: 819 GGAAGATGACAATCATCCTTTGAAAAATAATTTTGAACACCTGGCAATAACAGTGACTGATCTTTTTATTGGTGGGACAGAGTCAATGAGCACAACACTG AG

|||| ||| |||||| |||| ||| |||||||||||||||| | ||||||||| | ||| ||| ||| | |||| || | ||| ||||| || ||

Subject: 830 GGAAAATGCCAATCAGTGGATGAACTATACACTTGAACACCTGGCAATCATGGTGACTGATTTGTTTTTTGCTGGAATAGAGACAGTAAGCTCAACAATG AG

24. gi|293362473|ref|XM_002730139.1| PREDICTED: Rattus norvegicus cytochrome P450, 2c37, transcript variant 2 (Cyp2c37), mRNA

Length = 1739

Score = 170.8 bits (188), Expect = 2.5e-39

Identities = 162/207 (78%), Positives = 162/207 (78%), Gaps = 0/207 (0%)

Query: 714 TTATATTAAAAATTTCCTTTTGGAGAAAATAAGGGAACATAAAGAATCATTGGATGTTACAATTCCTCGGGACTTTATTGATTATTTCCTAATTAAAGGA

|||||||| ||| | |||| || ||||||||| |||||| |||||||| |||||||||||| ||||||||||||||||||||||| ||||||||| |

Subject: 720 TTATATTAGAAACTACCTTCTGAAGAAAATAAAAGAACATCAAGAATCACTGGATGTTACAAATCCTCGGGACTTTATTGATTATTACCTAATTAAGTGG

Query: 814 GCCCAGGAAGATGACAATCATCCTTTGAAAAATAATTTTGAACACCTGGCAATAACAGTGACTGATCTTTTTATTGGTGGGACAGAGTCAA~~TG~~AGCACAA

|||| | || |||||| | |||| || || |||| || | || | || ||||||||| | ||| || |||||||||| ||| |||||||

Subject: 820 AAGCAGGTAAATCACAATCCACATTTGGAATTTACACTTGAGAACTTATCAGTCACTGTGACTGATTTGTTTGGTGCTGGGACAGAGACAACAAGCACAA

Query: 914 CACTGAG

|||||||

Subject: 920 CACTGAG

25. gi|72679614|gb|BC100092.1| Rattus norvegicus cytochrome P450-like, mRNA (cDNA clone IMAGE:7372330), partial cds

Length = 1268

Score = 167.2 bits (184), Expect = 3e-38

Identities = 160/205 (78%), Positives = 160/205 (78%), Gaps = 0/205 (0%)

Query: 716 ATATTAAAAATTTCCTTTTGGAGAAAATAAGGGAACATAAAGAATCATTGGATGTTACAATTCCTCGGGACTTTATTGATTATTTCCTAATTAAAGGAGC

|||||| ||| | |||| || ||||||||| || ||| |||||||| |||||||||||| ||| ||||||||||||||||||| ||||||||| |

Subject: 353 ATATTAGAAACTACCTTCTGAAGAAAATAAAAGAGCATCAAGAATCACTGGATGTTACAAATCCCCGGGACTTTATTGATTATTACCTAATTAAGTGGAA

Query: 816 CCAGGAAGATGACAATCATCCTTTGAAAAATAATTTTGAACACCTGGCAATAACAGTGACTGATCTTTTTATTGGTGGGACAGAGTCAA~~TG~~AGCACAACA

|||||| || |||||| | || | || || ||||| || | |||| || ||||||||| | ||| || |||||||||| ||| |||||||||

Subject: 453 GCAGGAAAATCACAATCCACATTCGGAATTTACACTTGAAAACTTATCAATCACTGTGACTGATTTGTTTGGTGCTGGGACAGAGACAACAAGCACAACA

Query: 916 CTGAG

|||||

Subject: 553 CTGAG

27. gi|62078536|ref|NM_001013904.1| Rattus norvegicus cytochrome P450-like (LOC293989), mRNA

>gi|59808917|gb|BC089886.1| R. norvegicus cytochrome P450-like, mRNA (cDNA clone MGC:109053 IMAGE:7370532), complete cds

Length = 1681

Score = 167.2 bits (184), Expect = 3e-38

Identities = 161/207 (77%), Positives = 161/207 (77%), Gaps = 0/207 (0%)

Query: 714 TTATATTAAAAATTTCCTTTTGGAGAAAATAAGGGAACATAAAGAATCA~~T~~TGGATGTTACAATTCCTCGGGACTTTATTGATTATTTCCTAATTAAAGGA

||||| || ||| | |||| || ||||||||| |||||| |||||||| |||||||||||| ||||||||||||||||||||||| ||||||||| |

Subject: 724 TTATAATAGAAACTACCTTCTGAAGAAAATAAAAGAACATCAAGAATCACTGGATGTTACAAATCCTCGGGACTTTATTGATTATTACCTAATTAAGTGG

Query: 814 GCCCAGGAAGATGACAATCATCCTTTGAAAAATAATTTTGAACACCTGGCAATAACAGTGACTGATCTTTTTATTGGTGGGACAGAGTCAA~~TG~~AGCACAA

|||||| || |||||| || || || ||||| || | |||| || ||||||||| | ||| || |||||||||| ||| |||||||

Subject: 824 AAGCAGGAAAATCACAATCCAAATTCAGAATTTACACTTGAAAACTTATCAATCACTGTGACTGATTTGTTTGCTGCTGGGACAGAGACAACAAGCACAA

Query: 914 CACTGAG

|||||||

Subject: 924 CACTGAG

28. gi|203876|gb|K03501.1|RATCYPPBA Rat cytochrome P450 PB1 (PB1-2 allele) mRNA

Length = 1614

Score = 167.2 bits (184), Expect = 3e-38

Identities = 160/205 (78%), Positives = 160/205 (78%), Gaps = 0/205 (0%)

Query: 716 ATATTAAAAATTTCCTTTTGGAGAAAATAAGGGAACATAAAGAATCA~~T~~TGGATGTTACAATTCCTCGGGACTTTATTGATTATTTCCTAATTAAAGGAGC

|||||| ||| | |||| || ||||||||| || ||| |||||||| |||||||||||| ||| ||||||||||||||||||| ||||||||| |

Subject: 701 ATATTAGAAACTACCTTCTGAAGAAAATAAAAGAGCATCAAGAATCACTGGATGTTACAAATCCCCGGGACTTTATTGATTATTACCTAATTAAGTGGAA

Query: 816 CCAGGAAGATGACAATCATCCTTTGAAAAATAATTTTGAACACCTGGCAATAACAGTGACTGATCTTTTTATTGGTGGGACAGAGTCAA~~TG~~AGCACAACA

|||||| || |||||| | || | || || ||||| || | |||| || ||||||||| | ||| || |||||||||| ||| |||||||||

Subject: 801 GCAGGAAAATCACAATCCACATTCGGAATTTACACTTGAAAACTTATCAATCACTGTGACTGATTTGTTTGGTGCTGGGACAGAGACAACAAGCACAACA

Query: 916 CTGAG

|||||

Subject: 901 CTGAG

29. gi|203874|gb|M13711.1|RATCYPPB Rat cytochrome P450 PB1 (PB1 allele) mRNA

Length = 1605

Score = 167.2 bits (184), Expect = 3e-38

Identities = 160/205 (78%), Positives = 160/205 (78%), Gaps = 0/205 (0%)

Query: 716 ATATTAAAAATTTCCTTTTGGAGAAAATAAGGGAACATAAAGAATCA~~T~~TGGATGTTACAATTCCTCGGGACTTTATTGATTATTTCCTAATTAAAGGAGC

|||||| ||| | |||| || ||||||||| || ||| |||||||| |||||||||||| ||| ||||||||||||||||||| ||||||||| |

Subject: 701 ATATTAGAAACTACCTTCTGAAGAAAATAAAAGAGCATCAAGAATCACTGGATGTTACAAATCCCCGGGACTTTATTGATTATTACCTAATTAAGTGGAA

Query: 816 CCAGGAAGATGACAATCATCCTTTGAAAAATAATTTTGAACACCTGGCAATAACAGTGACTGATCTTTTTATTGGTGGGACAGAGTCAATGAGCACAACA

|||||| || |||||| | || | || || ||||| || | |||| || ||||||||| | ||| || |||||||||| ||| |||||||||

Subject: 801 GCAGGAAAATCACAATCCACATTCGGAATTTACACTTGAAAACTTATCAATCACTGTGACTGATTTGTTTGGTGCTGGGACAGAGACAACAAGCACAACA

Query: 916 CTGAG

|||||

Subject: 901 CTGAG

30. gi|203781|gb|M18336.1|RATCYP45R Rat cytochrome P450 processed pseudogene mRNA, complete cds

Length = 1636

Score = 167.2 bits (184), Expect = 3e-38

Identities = 160/205 (78%), Positives = 160/205 (78%), Gaps = 0/205 (0%)

Query: 716 ATATTAAAAATTTCCTTTTGGAGAAAATAAGGGAACATAAAGAATCA~~T~~TGGATGTTACAATTCCTCGGGACTTTATTGATTATTTCCTAATTAAAGGAGC

|||||| ||| | |||| || ||||||||| || ||| |||||||| |||||||||||| ||| ||||||||||||||||||| ||||||||| |

Subject: 736 ATATTAGAAACTACCTTCTGAAGAAAATAAAAGAGCATCAAGAATCACTGGATGTTACAAATCCCCGGGACTTTATTGATTATTACCTAATTAAGTGGAA

Query: 816 CCAGGAAGATGACAATCATCCTTTGAAAAATAATTTTGAACACCTGGCAATAACAGTGACTGATCTTTTTATTGGTGGGACAGAGTCAA~~TG~~AGCACAACA

|||||| || |||||| | || | || || ||||| || | |||| || ||||||||| | ||| || |||||||||| ||| |||||||||

Subject: 836 GCAGGAAAATCACAATCCACATTCGGAATTTACACTTGAAAACTTATCAATCACTGTGACTGATTTGTTTGGTGCTGGGACAGAGACAACAAGCACAACA

Query: 916 CTGAG

|||||

Subject: 936 CTGAG

31. gi|298880944|emb|FQ210718.1| Rattus norvegicus TL0ABA23YL12 mRNA sequence

Length = 1636

Score = 165.4 bits (182), Expect = 1e-37

Identities = 162/208 (77%), Positives = 162/208 (77%), Gaps = 1/208 (0%)

Query: 714 TTATATTAAAAATTTCCTTTTGGAGAAAATAAGGGAACATAAAGAATCATTGGATGTTACAATTCCTCGGGACTTTATTGATTATTTCCTAATTAAAGGA

||||| || ||| | |||| || ||||||||| |||||| |||||||| |||||||||||| ||||||||||||||||||||||| ||||||||| |

Subject: 733 TTATAATAGAAACTACCTTCTGAAGAAAATAAAAGAACATCAAGAATCACTGGATGTTACAAATCCTCGGGACTTTATTGATTATTACCTAATTAAGTGG

Query: 814 GCCCAGGAAGATGACAATCATCCTTTGA-AAAATAATTTTGAACACCTGGCAATAACAGTGACTGATCTTTTTATTGGTGGGACAGAGTCAA~~TG~~AGCACA

|||||| || ||||| ||| | || || ||||| || | |||| || ||||||||| | ||| || |||||||||| ||| ||||||

Subject: 833 AAGCAGGAAAATCCCAATCCAAATTTCAGAATTTACACTTGAAAACTTTTCAATCACTGTGACTGATTTGTTTGCTGCTGGGACAGAGACAACAAGCACA

Query: 913 ACACTGAG

||||||||

Subject: 933 ACACTGAG

32. gi|298913992|emb|FQ219720.1| Rattus norvegicus TL0ABA43YH14 mRNA sequence

Length = 1201

Score = 163.6 bits (180), Expect = 3.6e-37

Identities = 162/210 (77%), Positives = 162/210 (77%), Gaps = 0/210 (0%)

Query: 711 TGCTTATATTAAAAATTTCCTTTTGGAGAAAATAAGGGAACATAAAGAATCATTGGATGTTACAATTCCTCGGGACTT~~T~~ATTGATTATTTCCTAATTAAA

|||| |||||| | || ||||||| ||||||||| |||||| ||||||| ||||||||| ||| |||||||||||| |||||||| ||||||||| ||

Subject: 181 TGCTGCTATTAAGAGTTACCTTTTGAAGAAAATAAAAGAACATGAAGAATCGTTGGATGTTTCAAATCCTCGGGACTTCATTGATTACTTCCTAATTCAA

Query: 811 GGAGCCCAGGAAGATGACAATCATCCTTTGAAAAATAATTTTGAACACCTGGCAATAACAGTGACTGATCTTTTTATTGGTGG~~G~~ACAGAGTCAATGAGCA

|| |||||| ||| |||||| | |||| ||| ||||| |||||||| |||||| || | ||||||||||| |||||| || |||

Subject: 281 AGATGTCAGGAAAATGGCAATCAGCAGATGAACTATACACAAGAACATCTGGCAATCCTGGTGACTAATTTGTTTATTGGTGGAACAGAGACATCAAGCT

Query: 911 CAACACTGAG

||| |||||

Subject: 381 TAACGCTGAG

33. gi|298911403|emb|FQ219491.1| Rattus norvegicus TL0ABA44YO14 mRNA sequence

Length = 1611

Score = 163.6 bits (180), Expect = 3.6e-37

Identities = 162/210 (77%), Positives = 162/210 (77%), Gaps = 0/210 (0%)

Query: 711 TGCTTATATTAAAAATTTCCTTTTGGAGAAAATAAGGGAACATAAAGAATCATTGGATGTTACAATTCCTCGGGACTT~~T~~ATTGATTATTTCCTAATTAAA

|||| |||||| | || ||||||| ||||||||| |||||| ||||||| ||||||||| ||| |||||||||||| |||||||| ||||||||| ||

Subject: 586 TGCTGCTATTAAGAGTTACCTTTTGAAGAAAATAAAAGAACATGAAGAATCGTTGGATGTTTCAAATCCTCGGGACTTCATTGATTACTTCCTAATTCAA

Query: 811 GGAGCCCAGGAAGATGACAATCATCCTTTGAAAAATAATTTTGAACACCTGGCAATAACAGTGACTGATCTTTTTATTGGTGG~~G~~ACAGAGTCAATGAGCA

|| |||||| ||| |||||| | |||| ||| ||||| |||||||| |||||| || | ||||||||||| |||||| || |||

Subject: 686 AGATGTCAGGAAAATGGCAATCAGCAGATGAACTATACACAAGAACATCTGGCAATCCTGGTGACTAATTTGTTTATTGGTGGAACAGAGACATCAAGCT

Query: 911 CAACACTGAG

||| |||||

Subject: 786 TAACGCTGAG

34. gi|298906205|emb|FQ219038.1| Rattus norvegicus TL0ABA48YC15 mRNA sequence

Length = 1413

Score = 163.6 bits (180), Expect = 3.6e-37

Identities = 162/210 (77%), Positives = 162/210 (77%), Gaps = 0/210 (0%)

Query: 711 TGCTTATATTAAAAATTTCCTTTTGGAGAAAATAAGGGAACATAAAGAATCATTGGATGTTACAATTCCTCGGGACTT~~T~~ATTGATTATTTCCTAATTAAA

|||| |||||| | || ||||||| ||||||||| |||||| ||||||| ||||||||| ||| |||||||||||| |||||||| ||||||||| ||

Subject: 392 TGCTGCTATTAAGAGTTACCTTTTGAAGAAAATAAAAGAACATGAAGAATCGTTGGATGTTTCAAATCCTCGGGACTTCATTGATTACTTCCTAATTCAA

Query: 811 GGAGCCCAGGAAGATGACAATCATCCTTTGAAAAATAATTTTGAACACCTGGCAATAACAGTGACTGATCTTTTTATTGGTGG~~G~~ACAGAGTCAATGAGCA

|| |||||| ||| |||||| | |||| ||| ||||| |||||||| |||||| || | ||||||||||| |||||| || |||

Subject: 492 AGATGTCAGGAAAATGGCAATCAGCAGATGAACTATACACAAGAACATCTGGCAATCCTGGTGACTAATTTGTTTATTGGTGGAACAGAGACATCAAGCT

Query: 911 CAACACTGAG

||| |||||

Subject: 592 TAACGCTGAG

35. gi|298903412|emb|FQ209627.1| Rattus norvegicus TL0ABA41YH11 mRNA sequence

Length = 1171

Score = 163.6 bits (180), Expect = 3.6e-37

Identities = 162/210 (77%), Positives = 162/210 (77%), Gaps = 0/210 (0%)

Query: 711 TGCTTATATTAAAAATTTCCTTTTGGAGAAAATAAGGGAACATAAAGAATCATTGGATGTTACAATTCCTCGGGACTT~~T~~ATTGATTATTTCCTAATTAAA

|||| |||||| | || ||||||| ||||||||| |||||| ||||||| ||||||||| ||| |||||||||||| |||||||| ||||||||| ||

Subject: 150 TGCTGCTATTAAGAGTTACCTTTTGAAGAAAATAAAAGAACATGAAGAATCGTTGGATGTTTCAAATCCTCGGGACTTCATTGATTACTTCCTAATTCAA

Query: 811 GGAGCCCAGGAAGATGACAATCATCCTTTGAAAAATAATTTTGAACACCTGGCAATAACAGTGACTGATCTTTTTATTGGTGG~~G~~ACAGAGTCAATGAGCA

|| |||||| ||| |||||| | |||| ||| ||||| |||||||| |||||| || | ||||||||||| |||||| || |||

Subject: 250 AGATGTCAGGAAAATGGCAATCAGCAGATGAACTATACACAAGAACATCTGGCAATCCTGGTGACTAATTTGTTTATTGGTGGAACAGAGACATCAAGCT

Query: 911 CAACACTGAG

||| |||||

Subject: 350 TAACGCTGAG

36. gi|293344208|ref|XR_085719.1| PREDICTED: Rattus norvegicus cytochrome P450, family 2, subfamily c, polypeptide 77, pseudogene (Cyp2c77-ps), miscRNA

Length = 1498

Score = 163.6 bits (180), Expect = 3.6e-37

Identities = 159/205 (77%), Positives = 159/205 (77%), Gaps = 0/205 (0%)

Query: 716 ATATTAAAAATTTCCTTTTGGAGAAAATAAGGGAACATAAAGAATCATTGGATGTTACAATTCCTCGGGACTTTATTGATTATTTCCTAATTAAAGGAGC

|||||| ||| | |||| || ||||||||| || ||| |||||||| |||||||||||| ||||| ||||||||||||||||| ||||||||| |

Subject: 723 ATATTAGAAACTACCTTCTGAAGAAAATAAAAGAGCATCAAGAATCACTGGATGTTACAAATCCTCAGGACTTTATTGATTATTACCTAATTAAGTGGAA

Query: 816 CCAGGAAGATGACAATCATCCTTTGAAAAATAATTTTGAACACCTGGCAATAACAGTGACTGATCTTTTTATTGGTGGGACAGAGTCAA~~TG~~AGCACAACA

|||| | || |||||| | || | || || ||||| || | |||| || ||||||||| | ||| || |||||||||| ||| |||||||||

Subject: 823 GCAGGTAAATCACAATCCACATTCGGAATTTACACTTGAAAACTTATCAATCACTGTGACTGATTTGTTTGGTGCTGGGACAGAGACAACAAGCACAACA

Query: 916 CTGAG

|||||

Subject: 923 CTGAG

37. gi|59808902|gb|BC089790.1| Rattus norvegicus cytochrome P450, family 2, subfamily c, polypeptide 12, mRNA (cDNA clone MGC:108649 IMAGE:7371667), complete cds

Length = 1753

Score = 163.6 bits (180), Expect = 3.6e-37

Identities = 162/210 (77%), Positives = 162/210 (77%), Gaps = 0/210 (0%)

Query: 711 TGCTTATATTAAAAATTTCCTTTTGGAGAAAATAAGGGAACATAAAGAATCATTGGATGTTACAATTCCTCGGGACTT~~T~~ATTGATTATTTCCTAATTAAA

|||| |||||| | || ||||||| ||||||||| |||||| ||||||| ||||||||| ||| |||||||||||| |||||||| ||||||||| ||

Subject: 719 TGCTGCTATTAAGAGTTACCTTTTGAAGAAAATAAAAGAACATGAAGAATCGTTGGATGTTTCAAATCCTCGGGACTTCATTGATTACTTCCTAATTCAA

Query: 811 GGAGCCCAGGAAGATGACAATCATCCTTTGAAAAATAATTTTGAACACCTGGCAATAACAGTGACTGATCTTTTTATTGGTGG~~G~~ACAGAGTCAATGAGCA

|| |||||| ||| |||||| | |||| ||| ||||| |||||||| |||||| || | ||||||||||| |||||| || |||

Subject: 819 AGATGTCAGGAAAATGGCAATCAGCAGATGAACTATACACAAGAACATCTGGCAATCCTGGTGACTAATTTGTTTATTGGTGGAACAGAGACATCAAGCT

Query: 911 CAACACTGAG

||| |||||

Subject: 919 TAACGCTGAG

38. gi|13994165|ref|NM_031572.1| Rattus norvegicus cytochrome P450, family 2, subfamily c, polypeptide 12 (Cyp2c12), mRNA

>gi|203681|gb|J03786.1|RATCY45FS Rat cytochrome P-450 (female-specific and growth hormone-inducible) mRNA, complete cds

Length = 1714

Score = 163.6 bits (180), Expect = 3.6e-37

Identities = 162/210 (77%), Positives = 162/210 (77%), Gaps = 0/210 (0%)

Query: 711 TGCTTATATTAAAAATTTCCTTTTGGAGAAAATAAGGGAACATAAAGAATCATTGGATGTTACAATTCCTCGGGACTT~~T~~ATTGATTATTTCCTAATTAAA

|||| |||||| | || ||||||| ||||||||| |||||| ||||||| ||||||||| ||| |||||||||||| |||||||| ||||||||| ||

Subject: 711 TGCTGCTATTAAGAGTTACCTTTTGAAGAAAATAAAAGAACATGAAGAATCGTTGGATGTTTCAAATCCTCGGGACTTCATTGATTACTTCCTAATTCAA

Query: 811 GGAGCCCAGGAAGATGACAATCATCCTTTGAAAAATAATTTTGAACACCTGGCAATAACAGTGACTGATCTTTTTATTGGTGG~~G~~ACAGAGTCAATGAGCA

|| |||||| ||| |||||| | |||| ||| ||||| |||||||| |||||| || | ||||||||||| |||||| || |||

Subject: 811 AGATGTCAGGAAAATGGCAATCAGCAGATGAACTATACACAAGAACATCTGGCAATCCTGGTGACTAATTTGTTTATTGGTGGAACAGAGACATCAAGCT

Query: 911 CAACACTGAG

||| |||||

Subject: 911 TAACGCTGAG

**EXAMPLES OF RECORDS WITH SOME HOMOLOGY BUT NO SECOND FLANKING REGION:**

190. gi|293344727|ref|XM_001080345.2| PREDICTED: Rattus norvegicus cytochrome P450, family 2, subfamily c, polypeptide 79 (Cyp2c79), partial mRNA

Length = 961

Score = 82.4 bits (90), Expect = 9.4e-13

Identities = 143/205 (69%), Positives = 143/205 (69%), Gaps = 10/205 (4%)

Query: 721 AAAAATTTCCTTTTGGAGAAAATAAGGGAACATAAAGAATCATTGGATGTTACAATTCCTCGGGACTTTATTGATTATTTCCTAATTA--AAGGAGCCCA

||| ||| | |||||| ||| ||| | ||| |||| ||| ||||| ||| | |||||||||||||||||||| |||||| || | | |||| ||

Subject: 721 AAACATTACTTTTTGGCCAAAGTAATACAGCATCAAGAGTCACTGGATATTAACAATCCTCGGGACTTTATTGATTGTTTCCTGATCAAGATGGAG--CA

Query: 819 GGAAGATGACAATCATCCTTTGAAAAA---TAATTTTGAACACCTGGCAATAACAGTGACTGATCTTTTTATTGGTGGGACAGAGTCAATGAGCACAACA

|||| | ||| | || || | | || || ||| || || | |||| ||||||| ||| || |||||||||| ||| || |||||

Subject: 819 GGAAAAACACA---ACCCAAAGACAGAGTTTACTTGTGAGAACTTGATCTTCACAGCATCTGATCTCTTTGCTGCTGGGACAGAGACAACTAGTACAACC

Query: 916 CTGAG

|||||

Subject: 916 CTGAG

194. gi|117558352|gb|BC127503.1| Rattus norvegicus cytochrome P450, family 2, subfamily c, polypeptide 7, mRNA (cDNA clone MGC:156667 IMAGE:7371672), complete cds

Length = 1808

Score = 80.6 bits (88), Expect = 3.3e-12

Identities = 141/200 (70%), Positives = 141/200 (70%), Gaps = 24/200 (12%)

Query: 733 TTGGAGAAAATAAGGGAACATAAAGAATCATTGGATGTTACAATTCCTCGGGACTTTATTGATTATTTCCTAATTAAAGGAGCCCAGGAAGATGACAATC

||| |||||||| ||||| | ||||||||||||||||||| ||||| || ||| ||||||||| ||| |||||| | |||| | |||| |

Subject: 793 TTGAAGAAAATAGAAGAACACCAGGAATCATTGGATGTTACAAACCCTCGTGATTTTGTTGATTATTACCTGATTAAACAAAAACAGGCA---AACAA-C

Query: 833 ATCCTTTGAAAAAT---AATTT-----TGAACACCTG----GCAATAACAGTGACTGATCTTTTTATTGGTGGGACAGAGTCAATGAGCACAACACTGAG

||| ||| ||| ||| | |||| | ||| ||| || || || ||||| || || ||||||||| |||||||||||||| ||||

Subject: 889 ATC----GAACAATCAGAATATTCACATGAAAATCTGACATGCAGTATCA-TG---GATCTCATTGGTGCAGGGACAGAGACAATGAGCACAACATTGAG
